# Comparison of the Effects of Brominated Perylenediimide and Perylene Tetraester Modified G‐C₃N₄ S‐Scheme Nanocomposites on the Photocatalytic Degradation of Anionic and Cationic Dyes and Herbicide

**DOI:** 10.1002/gch2.202500100

**Published:** 2025-04-27

**Authors:** Erkan Aksoy, Emre Alp

**Affiliations:** ^1^ Project and Technology Office, Rectorate Bartin University Bartin 74100 Türkiye; ^2^ Department of Metallurgy and Materials Engineering Faculty of Engineering Architecture and Design Bartın University Bartin Türkiye

**Keywords:** g‐C₃N₄, metal‐free photocatalyst, nanomaterials, perylene tetraester, perylenediimide, S‐scheme heterojunction, ultrasonic self‐assembly3

## Abstract

Metal‐free g‐C_3_N_4_ (graphitic carbon nitride) is a promising candidate for the next‐generation visible light‐responsive photocatalyst; however, the recombination and transfer of the photogenerated charge carriers restrict its photocatalytic performances. The exfoliated g‐C₃N₄ sensitized with brominated perylenediimide (dBrPDI) and perylene tetraester (dBrPTE) enhances the photocatalytic performance due to improved charge separation, light absorption, charge transfer and, thereby, overall efficiency in pollutant degradation. The g‐C_3_N_4_/dBrPTE hybrid composite exhibits the fastest photocatalytic degradation against rhodamine B (RhB) pollutants. The g‐C_3_N_4_/dBrPTE hybrid composite degrades RhB with a 2.34‐fold improvement over pure g‐C_3_N_4_, while the g‐C_3_N_4_/dBrPDI hybrid composite degrades with a 1.56‐fold increase over pure g‐C_3_N_4_. The g‐C3N4/dBrPDI hybrid composite shows the highest photocatalytic efficiency against methyl orange (MO) pollutants. The g‐C_3_N_4_/dBrPDI hybrid composite degrades MO with a 2.25‐fold improvement over pure g‐C_3_N_4_, while the g‐C_3_N_4_/dBrPTE hybrid composite degrades with a 1.8‐fold increase over pure g‐C_3_N_4_. Unlike MO and RhB, the perylene dye sensitization does not enhance the photocatalytic degradation of 2,4‐dichloro phenoxy acetic acid (2,4‐D) and no sustained increase in efficiency is not observed. Overall, these results suggest that photocatalytic efficiency depends not only on the sensitized photocatalyst material but also on the interaction between the sensitized photocatalyst and the chemical and ionic properties of the pollutants in the aquatic media.

## Graphical abstract

Exfoliated g‐C₃N₄ sensitized with brominated perylene dyes (dBrPDI and dBrPTE) significantly enhances photocatalytic degradation of RhB and methyl orange via improved charge separation and light absorption. Performance varies by pollutant type, highlighting that photocatalytic efficiency is strongly influenced by dye–pollutant interactions in aqueous environments.

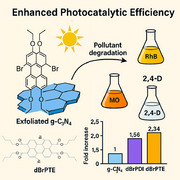

## 1 Introduction

Environmental pollution, particularly water pollution, poses significant challenges to ecosystems and human health. Water bodies are increasingly contaminated by persistent organic pollutants (POPs), which are resistant to conventional treatment methods and can bioaccumulate in the food chain, leading to severe ecological and health impacts.^[^ ^1^ ^]^ These carcinogenic and toxic POPs pose a significant environmental threat to the global water cycle, making it imperative to remove them from wastewater. Various methods have been developed and optimized to treat wastewater effectively, categorized broadly into physical, chemical, and biological processes, such as biodegradation, coagulation, adsorption, membrane process, advanced oxidation process (AOP). Each method has its advantages and limitations, making it essential to select appropriate techniques based on specific wastewater characteristics and treatment goals.^[^ ^2^ ^]^ AOPs have emerged as promising technologies for the remediation of such pollutants. AOPs utilize reactive oxygen species (ROS), primarily hydroxyl radicals (•OH), to nonselectively degrade organic contaminants into less harmful substances, such as carbon dioxide and water.^[^ ^3^, ^4^ ^]^

Heterogeneous photocatalysis, a subset of AOPs, involves the use of semiconductor materials to facilitate the generation of reactive species under light irradiation. This method has gained traction due to its effectiveness in degrading a wide range of organic pollutants, including dyes, pharmaceuticals, and pesticides.^[^ ^5^ ^]^ Compared to conventional treatment methods, the heterogeneous photocatalytic process offers several key benefits: it efficiently utilizes renewable solar energy, achieves rapid oxidation with minimal energy requirements, achieves rapid oxidation with minimal energy requirements, and operates under milder reaction conditions.^[^ ^6^ ^]^ Heterogeneous photocatalysis is a promising technology for environmental remediation, particularly in the degradation of organic pollutants. However, it faces several significant drawbacks that can hinder its efficiency and practical application. The main drawbacks of heterogeneous photocatalysis are limited light absorption, which benefits from only a tiny fraction of the solar spectrum and the rapid recombination of photogenerated electron–hole pairs, reducing the availability of charge carriers for the desired oxidation and reduction reactions, thereby lowering the overall photocatalytic efficiency.^[^ ^7^, ^8^ ^]^ To overcome the limitation of UV light absorption, researchers are focusing on developing photocatalysts that can utilize visible light more effectively.^[^ ^9^, ^10^, ^11^ ^]^ Creating nanostructured photocatalysts or nanocomposites can significantly increase the surface area and enhance charge separation. For instance, combining different semiconductor materials can create heterojunctions that facilitate charge transfer and reduce recombination rates.^[^ ^12^, ^13^ ^]^

Graphitic carbon nitride (g‐C₃N₄) has gained significant attention as a photocatalyst due to its unique properties and advantages in various photocatalytic applications, including water splitting, CO₂ reduction, and organic pollutant degradation. A metal‐free nature, chemical and thermal stability, nontoxic, visible light absorption, rich surface properties, 2D structure, and facile synthesis are the key properties of g‐C₃N₄ that make it a preferred material for photocatalysis processes.^[^ ^14^, ^15^, ^16^, ^17^, ^18^ ^]^ Bulk g‐C₃N₄ can be easily synthesized through the thermal polymerization of precursors, such as urea, cyanamide, dicyandiamide, or melamine.^[^ ^19^, ^20^, ^21^, ^22^, ^23^, ^24^, ^25^ ^]^ However, bulk g‐C₃N₄ has a small specific surface area, and the photogenerated charge carriers tend to recombine, which negatively affects the photocatalytic processes that occur at the g‐C₃N₄ surface.

In recent years, various strategies have been developed to improve the photocatalytic activity of g‐C_3_N_4_. The photocatalytic activity of g‐C₃N₄ can be significantly enhanced through various strategies, including heteroatom doping, heterojunction formation, nanocomposite integration, morphological modifications, surface modification and defect engineering, and quantum dot incorporation.^[^ ^26^, ^27^, ^28^, ^29^, ^30^ ^]^ These approaches not only improve the light absorption and charge separation efficiency of g‐C₃N₄ but also increase the number of active sites available for photocatalytic reactions. In light of these strategies, g‐C₃N₄ has been shown to utilize the solar spectrum better, effectively separate and transfer photogenerated electrons and holes, and increase the efficiency of reactive oxygen species in initiating photocatalytic reactions.^[^ ^31^, ^32^, ^33^, ^34^ ^]^

Perylene diimides (PDIs) are a class of organic compounds that have garnered significant attention in the fields of organic electronics due to their exceptional electronic properties, thermal stability, and versatility in various applications. Structurally, PDIs are characterized by a perylene core with two imide groups at the 3,4 and 9,10 positions, which contribute to their strong electron‐accepting capabilities and high electron mobility.^[^ ^35^ ^]^ The ability to tailor the chemical structure of PDIs has led to their use in a wide range of applications, including organic photovoltaics, organic light‐emitting diodes (OLEDs), and fluorescent probes in biological imaging.^[^ ^36^ ^]^ The most notable features of PDIs are their strong light absorption and efficient photon‐energy transfer abilities, which make them ideal for applications in photophysics and electronics.^[^ ^37^, ^38^, ^39^ ^]^ The unique properties of PDIs, including their ability to form π–π stacking interactions and their high electron affinities, have made them a focal point in developing advanced materials for organic electronics and optoelectronic devices.^[^ ^40^ ^]^ In recent years, the incorporation of PDIs into nanocomposite architectures has further enhanced their photocatalytic activity and efficiency in energy conversion processes. For instance, PDIs have been integrated into S‐scheme heterojunctions, which facilitate effective charge separation and enhance the generation of reactive oxygen species (ROS) during photocatalytic reactions. This capability is particularly valuable in environmental applications, such as the degradation of organic pollutants and the reduction of carbon dioxide.^[^ ^41^ ^]^

Perylene tetraesters (PTEs) represent a significant class of compounds in organic chemistry due to their unique structural and electronic properties. The fundamental importance of these compounds lies in their π‐conjugated systems, which facilitate strong electronic interactions and contribute to their remarkable optical characteristics. The molecular structure of perylene tetraesters, characterized by a planar configuration and extensive conjugation, enhances their ability to absorb and emit light efficiently, making them suitable for various applications in organic electronics and photonics.^[^ ^42^, ^43^, ^44^, ^45^, ^46^ ^]^ The π‐conjugated system of perylene tetraesters is pivotal in defining their optical and electronic properties. The extended conjugation allows for effective delocalization of π‐electrons, resulting in high fluorescence quantum yields and strong absorption in the visible spectrum.^[^ ^47^, ^48^ ^]^ The molecular structure can be modified through functionalization at various positions, which can tailor the electronic properties and enhance the performance of devices.^[^ ^49^, ^50^ ^]^ The introduction of electron‐donating or electron‐withdrawing groups can significantly alter the energy levels of the highest occupied molecular orbital (HOMO) and lowest unoccupied molecular orbital (LUMO), thus optimizing the charge transport properties in electronic applications.^[^ ^51^, ^52^ ^]^

Various methods, such as using local surface plasmon resonance (LSPR) effects and constructing heterostructures, are utilized to achieve higher photocatalytic performances.^[^ ^53^, ^54^ ^]^ The quest for efficient photocatalysts has led to various heterojunction architectures, among which the S‐scheme heterojunction has emerged as a promising strategy to enhance photocatalytic performance. This innovative approach leverages the unique charge transfer mechanisms inherent to S‐scheme configurations, which effectively separate and utilize photogenerated charge carriers.^[^ ^55^ ^]^ In an S‐scheme heterojunction, two semiconductors are combined in such a way that the conduction band (CB) of one semiconductor is aligned with the valence band (VB) of the other, creating a spatially separated charge transfer pathway. This configuration allows for the efficient utilization of both electrons and holes, leading to improved redox capabilities and enhanced photocatalytic activity.^[^ ^56^ ^]^ Unlike traditional Z‐scheme or type‐II heterojunctions, the S‐scheme design minimizes charge recombination by ensuring that the photogenerated electrons and holes are retained in their respective semiconductors, thus maximizing their participation in photocatalytic reactions.^[^ ^57^, ^58^ ^]^ PTEs and PDIs have garnered significant attention in photocatalytic applications, particularly when combined with graphitic carbon nitride (g‐C₃N₄) to form heterostructures. In recent years, organic dyes have attracted increasing interest not only due to their cost‐effectiveness but also owing to their environmentally benign characteristics.^[^ ^59^, ^60^ ^]^ Among these, perylene‐based derivatives are particularly notable for their high molar extinction coefficients (*ε*) in the visible spectral region, as well as their exceptional photostability and thermal stability in both solution and solid‐state forms.^[^ ^61^, ^62^, ^63^ ^]^ In photocatalytic applications, efficient absorption of visible light is a pivotal factor for enhancing catalytic activity. Spectral analysis reveals that dBrPDI exhibits strong absorption in the 462–528 nm range, while dBrPTE displays effective absorption between 445 and 468 nm, highlighting their suitability for visible‐light‐driven photocatalytic systems.^[^ ^64^ ^]^ Moreover, perylene derivatives exhibit strong π–π stacking arising from their extended conjugation systems and planar structures.^[^ ^65^ ^]^ The researches underscore the importance of π–π interactions in the behavior of perylene derivatives when integrated into polymer matrices. The perylene derivative is noted for its structural properties, which may facilitate π–π stacking with itself or other materials if utilized in composite structures, including g‐C₃N₄.^[^ ^66^ ^]^ The unique optical properties of perylene derivatives, combined with the electronic characteristics of g‐C₃N₄, make these materials suitable for use in solar energy conversion systems.^[^ ^67^, ^68^ ^]^ The incorporation of PTEs and PDIs into g‐C₃N₄ heterostructures improves charge separation and charge recombination rates, thereby enhancing the overall photocatalytic activity.^[^ ^69^ ^]^

**Figure 1:**
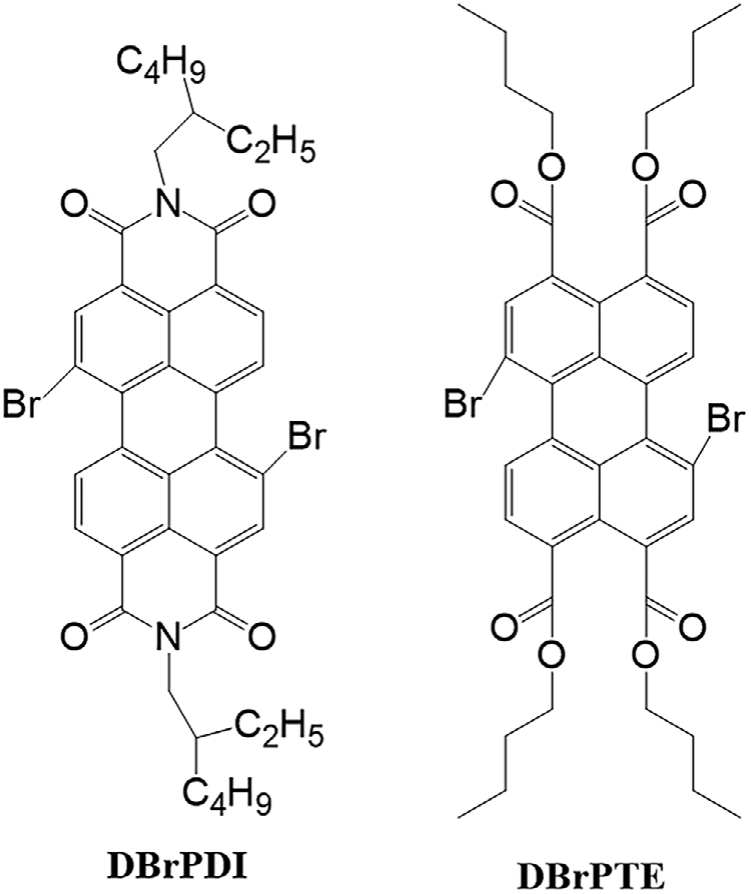
Molecular structures of perylene derivatives used as sensitizers. The molecule on the left is dBrPDI, and the molecule on the right is dBrPTE.

Herein, the visible‐responsive graphitic carbon nitride‐based (g‐C₃N₄) sensitized with perylene diimide (g‐C_3_N_4_/dBrPDI) and perylene tetraester (g‐C_3_N_4_/dBrPTE) heterojunction nanocomposites are elaborately developed for effective pollutant degradation in photocatalytic processes. The S‐scheme g‐C₃N₄ sensitized with perylene derivations hybrid nanocomposites are constructed through ultrasonic‐assisted self‐assembly. The photocatalytic activities of hybrid nanocomposites are conducted on artificial dyes (methyl orange and rhodamine B), representing anionic and cationic characteristics and herbicide (2,4‐dichloro phenoxy acetic acid), which is one of the oldest and most widely used chemicals for agricultural activities in the world. The well‐designed perylene derivatives sensitized graphitic carbonitride‐based hybrid nanocomposites exhibit exceptional photocatalytic performance, which could be attributed to the constructed S‐scheme heterojunction with efficient electron transfer. The interfacial π–π interactions between graphitic carbonitride and perylene derivatives would be capable of not only contributing to the adhesion of perylene derivatives with the graphitic carbonitride but also supporting the interface charge transfer and charge separation mechanisms under the S‐scheme construction. This work contributes to the design of S‐scheme‐constructed photocatalysts to overcome charge transfer and separation issues using the g‐C_3_N_4_‐based heterojunction nanocomposites for efficient photocatalytic degradation of pollutants.(**Figure** **1**)

## 2 Results and Discussion

**Figure 2:**
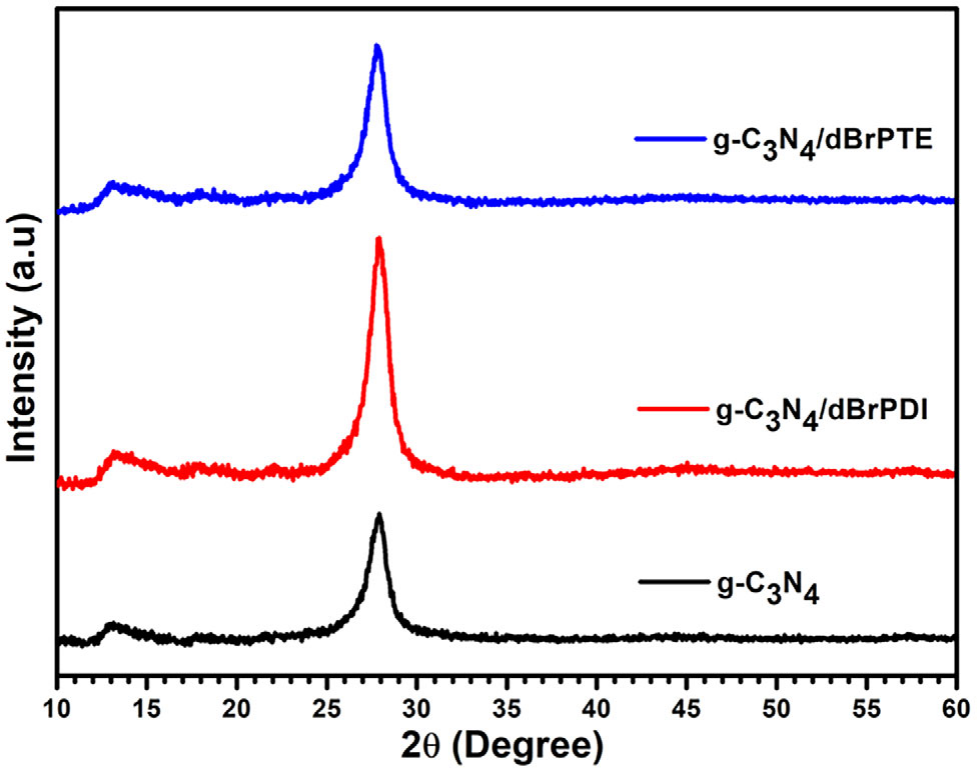
XRD patterns of the exfoliated g‐C_3_N_4_ nanosheets, dBrPDI sensitized, and dBrPTE sensitized g‐C_3_N_4_ hybrid nanocomposites.

**Figure 3:**
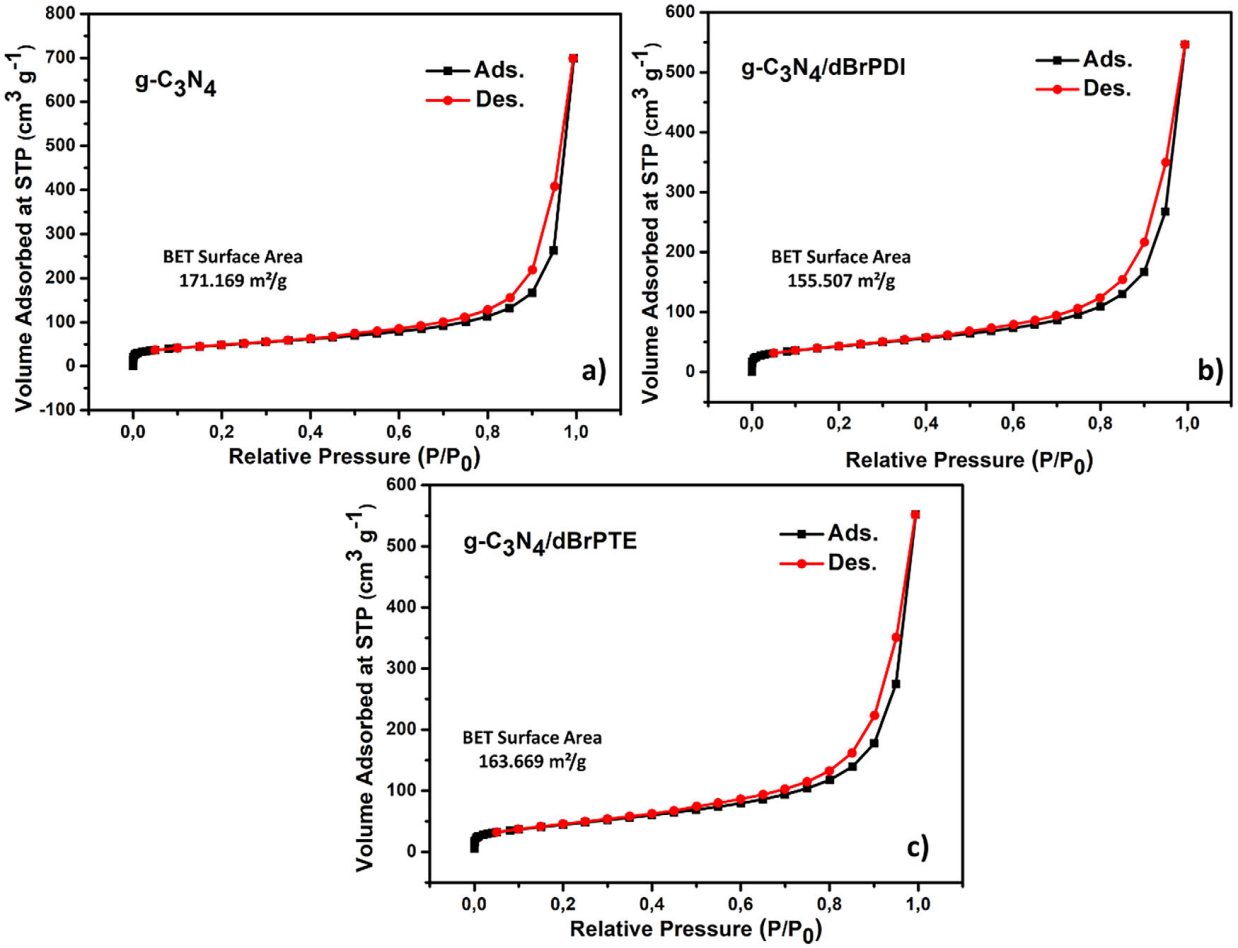
Brunauer–Emmett–Teller (BET) nitrogen adsorption–desorption isotherm curves for a) pure g‐C_3_N_4_ nanosheets obtained by thermal exfoliation process and b) g‐C_3_N_4_/dBrPDI hybrid nanocomposite and c) g‐C_3_N_4_/dBrPTE hybrid nanocomposite.

In the beginning, we will characterize and present the features of the exfoliated g‐C_3_N_4_ and g‐C_3_N_4_ sensitized by dBrPDI and dBrPTE to have better knowledge and insight into their main photocatalytic activities. The X‐ray diffraction patterns of synthesized samples are presented in **Figure** **2**. Two characteristic diffraction peaks corresponding to the structure of g‐C_3_N_4_ are observed. The peak at 27.4° corresponds to the (002) interlayer diffraction, which is attributed to the stacking of 2D CN layers in a graphite‐like layered structure. The diffraction peak at 13° is associated with the (100) planes, which are related to the in‐plane arrangement of tri‐*s*‐triazine units.^[^ ^72^, ^73^ ^]^ The peaks corresponding to perylene derivatives are not observed in the X‐ray reflections of dBrPTE and dBrPDI sensitized g‐C_3_N_4_, indicating the trace amount of the derivatives attached to the surface of g‐C_3_N_4_ nanosheets as well. It is observed that the intensity of the diffraction peaks associated with the (002) planes in the hybrid nanocomposites sensitized with perylene derivatives changes relative to pure g‐C_3_N_4_ nanosheets. It is observed that the reflection peaks, especially (002), in the hybrid nanocomposite sensitized with dBrPTE and dBrPDI derivatives, show higher intensities than pure g‐C_3_N_4_. In the thermal oxidation process for exfoliating layers, the planes resulting from the stacking of 2D CN layers are expectedly fragmented, leading to a decrease in crystalline ordering, as seen in the reduced peak intensities in the X‐ray diffractometer (XRD). The X‐ray reflection peaks corresponding to the (002) planes in the hybrid nanocomposites sensitized with dBrPTE and dBrPDI of perylene derivatives show higher intensities than bare g‐C_3_N_4_ nanoparticles exposed to the thermal exfoliation process. Accordingly, it is thought that the sensitization process with perylene causes the planes to restick to each other and become more regular. It is thought that the weak XRD peaks of pure g‐C_3_N_4_ to the disturbance of the graphitic‐like structure are attributed to the enlarged surface area. This phenomenon is consistent with the surface area results obtained from Brunauer–Emmett–Teller (BET) measurements, which are given in **Figure** **3**.

In Figure 2, the surface area analyses of synthesized pure g‐C_3_N_4_, g‐C_3_N_4_/dBrPDI, and g‐C_3_N_4_/dBrPTE hybrid nanocomposites are presented. The pure g‐C_3_N_4_ obtained by thermal exfoliation process exhibits the highest surface area, ≈171 m^2^ g^−1^. The g‐C_3_N_4_/dBrPTE hybrid nanocomposite shows a total surface area of ≈164 m^2^ g^−1^, while the g‐C_3_N_4_/dBrPDI hybrid nanocomposite exhibits a total surface area of ≈156 m^2^ g^−1^. As expected, the thermal exfoliation process results in the separation of graphitic‐like layers, leading to an increase in surface area due to the separation between these layers. Weak and broad XRD peaks of the (002) plane at 27.4° indicate the disturbance of the graphitic structure, which is associated with an enlarged surface area and the presence of nanolayers.^[^ ^74^ ^]^ The nanoparticles with the highest surface area belong to the pure g‐C_3_N_4_ obtained by thermal exfoliation treatment (≈171 m^2^ g^−1^) and accordingly, it has the lowest peak intensities. The total surface area of the g‐C_3_N_4_ nanocomposite sensitized with dBrPTE (g‐C_3_N_4_/dBrPTE) is measured as ≈164 m^2^ g^−1^, and the total surface area of the nanocomposite sensitized with dBrPDI (g‐C_3_N_4_/dBrPDI) is ≈156 m^2^ g^−1^. While the most intense peak in the X‐ray diffraction reflections is observed for the g‐C_3_N_4_/dBrPDI nanocomposite, it has the lowest total surface area. Similarly, the X‐ray diffraction intensity of the g‐C_3_N_4_/dBrPTE nanocomposite is between the intensities of the other two nanocomposites, and its total surface area is also between those of the other two structures. When the X‐ray diffraction and BET results are considered together, it is evident that the thermal exfoliation treatment caused significant exfoliation of the layers; however, the sensitization process with perylene derivatives led to the reaggregation of the layers, restoring some of the structural layer order. As a result of this physical phenomenon, it is observed that increased structural order between the layers leads to more intense peaks in the XRD results and relatively lower total surface area in the BET results for the nanocomposite structures. It is suggested that the pi–pi stacking interactions resulting from the sensitization process with perylene derivatives and/or ultrasonic‐assisted self‐assembly method lead to the reaggregation of the separated graphitic‐like layers, resulting in a reduction in surface area.

**Figure 4:**
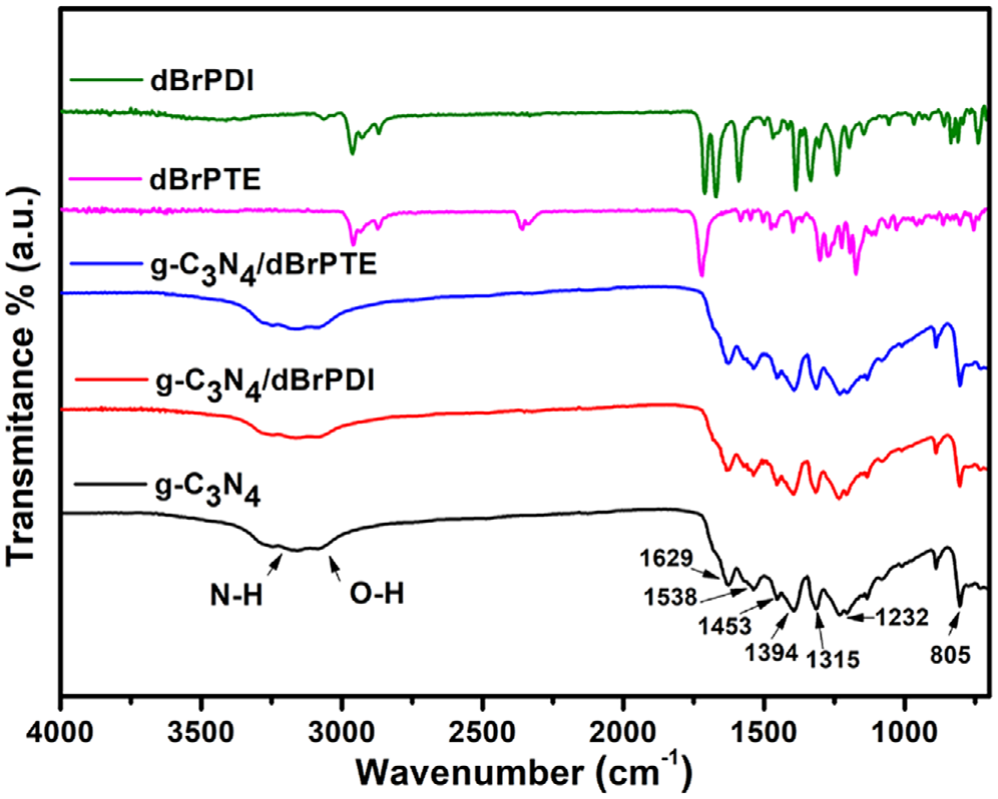
Fourier transform infrared (FTIR) spectroscopy spectra of g‐C_3_N_4_, g‐C_3_N_4_/dBrPDI ve g‐C_3_N_4_/dBrPTE nanocomposites, dBrPDI and dBrPTE dyes.

Fourier transform infrared (FTIR) spectra of the synthesized perylenes derivatives and the produced hybrid nanocomposite are presented in **Figure** **4**. The strong bands observed between 1200 and 1650 cm^−1^ correspond to the stretching vibrations of the heptazine heterocyclic ring (C_6_N_7_) units, while the sharp absorption peak at 805 cm^−1^ is associated with their breathing modes.^[^ ^75^, ^76^ ^]^ The broad absorption band in the 2900–3300 cm^−1^ region is attributed to the adsorption of water (O*─*H stretching mode) and the presence of uncondensed amine groups (N*─*H stretching mode).^[^ ^77^, ^78^ ^]^ The well‐resolved spectrum in g‐C_3_N_4_ exhibits firm absorption peaks between 1629 and 1232 cm^−1^ and the ring breath at cm^−1^, suggesting that the polymeric material is fairly well‐ordered on a molecular level.^[^ ^79^ ^]^

The FTIR spectrum represented by the purple curve in Figure 3 shows absorption peaks at 2956 cm^−1^ (C_Ar_ *─*H), 2869 cm^−1^ (C*─*H), 1717 cm^−1^ (C═O), and 1300–1175 cm^−1^ (C*─*O), characterizing 1,7‐dibromoperylene‐3,4,9,10‐tetracarboxy tetrabutyl ester (dBrPTE). The FTIR spectrum depicted by the green curve shows peaks at 2960 and 2928 cm^−1^ (C_Ar_ *─*H), 2866 cm^−1^ (C*─*H), 1709 and 1669 cm^−1^ (C═O), 1588 cm^−1^ (C_Ar_ *─*C_Ar_), 1386 and 1333 cm^−1^ (HC═C), and 1242 cm^−1^ (C*─*N), corresponding to *N*,*N*′‐Bis(2,6‐diisopropylphenyl)‐dibromoperylene‐3,4,9,10‐tetracarboxybisimide (dBrPDI).^[^ ^62^ ^]^ No significant shift in absorption bands corresponding with g‐C_3_N_4_ was observed compared to pure g‐C_3_N_4_ in analysis of the FTIR spectra of perylene derivatives sensitized g‐C_3_N_4_ hybrid nanocomposites. The absence of shift can be attributed to the trace amount of perylene dye derivatives in the hybrid nanocomposite, resulting in no noticeable changes in the FTIR spectra.

**Figure 5:**
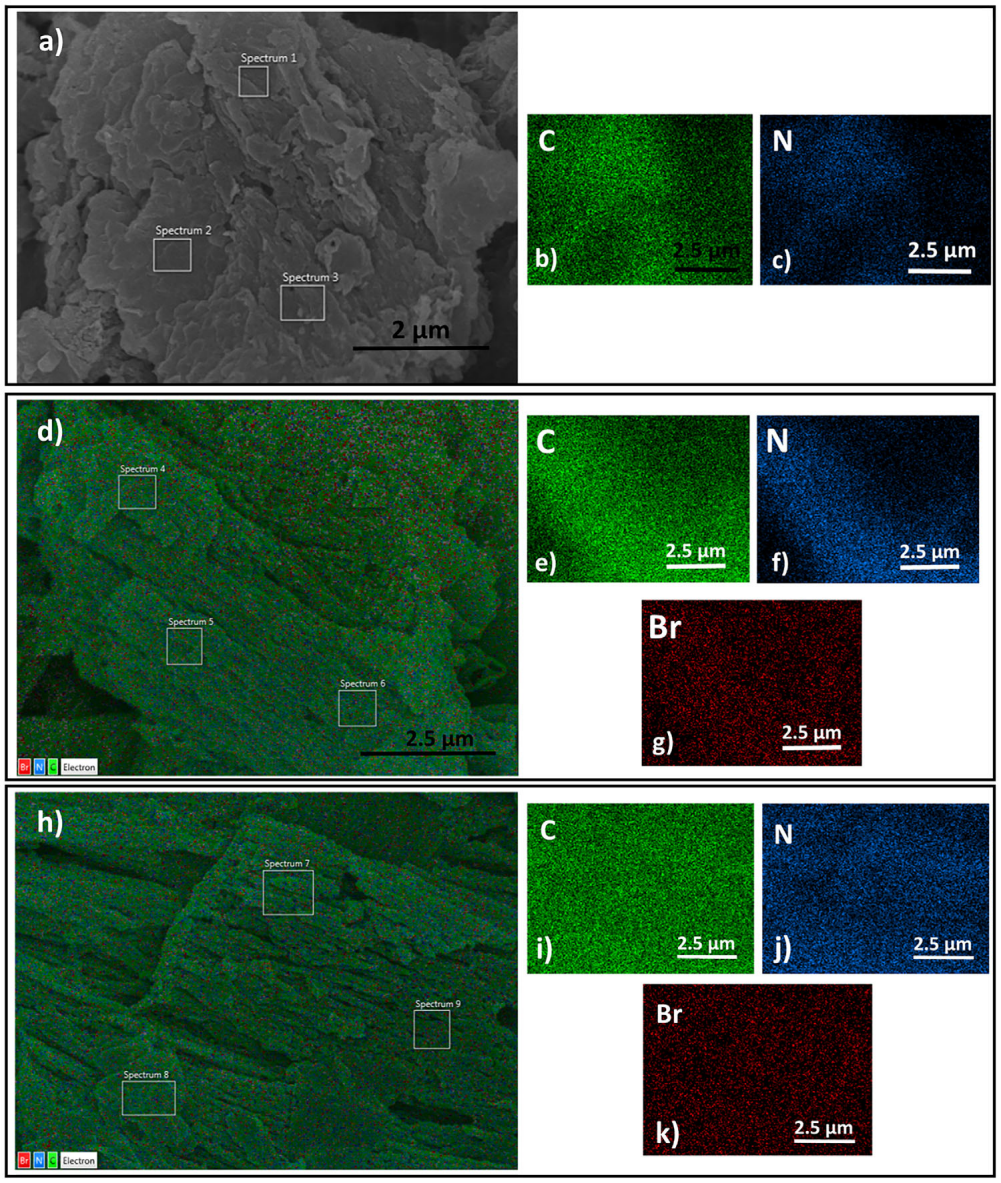
Field emission scanning electron microscopy (FESEM) images of g‐C_3_N_4_, g‐C_3_N_4_/dBrPDI ve g‐C_3_N_4_/dBrPTE nanocomposites along with energy dispersive X‐ray spectroscopy (EDS) elemental mapping analysis.

**Figure** **5** represents field emission scanning electron microscopy (FESEM) images of g‐C_3_N_4_, g‐C_3_N_4_/dBrPDI ve g‐C_3_N_4_/dBrPTE nanocomposites along with energy dispersive X‐ray spectroscopy (EDS) elemental mapping analysis. The FESEM images show that nanoparticles exhibit a typical morphology of g‐C_3_N_4_. It is observed that the sensitization process with perylene derivatives does not have any significant effect on the morphology of g‐C_3_N_4_. The presence of bromine in the perylene derivatives is detected in the elemental spectra of the hybrid composites from the EDS elemental mapping analyses, given in Figure 4d–k. Accordingly, as seen in FESEM images of perylene derivatives sensitized hybrid nanocomposite (Figure 4d–k), the homogeneously dispersed bromine within the nanocomposite hybrid structure (an atomic 0.2%, indicates that the perylene dye derivatives are successfully attached to the g‐C_3_N_4_ surfaces through an ultrasonic‐assisted self‐assembly method.

**Figure 6:**
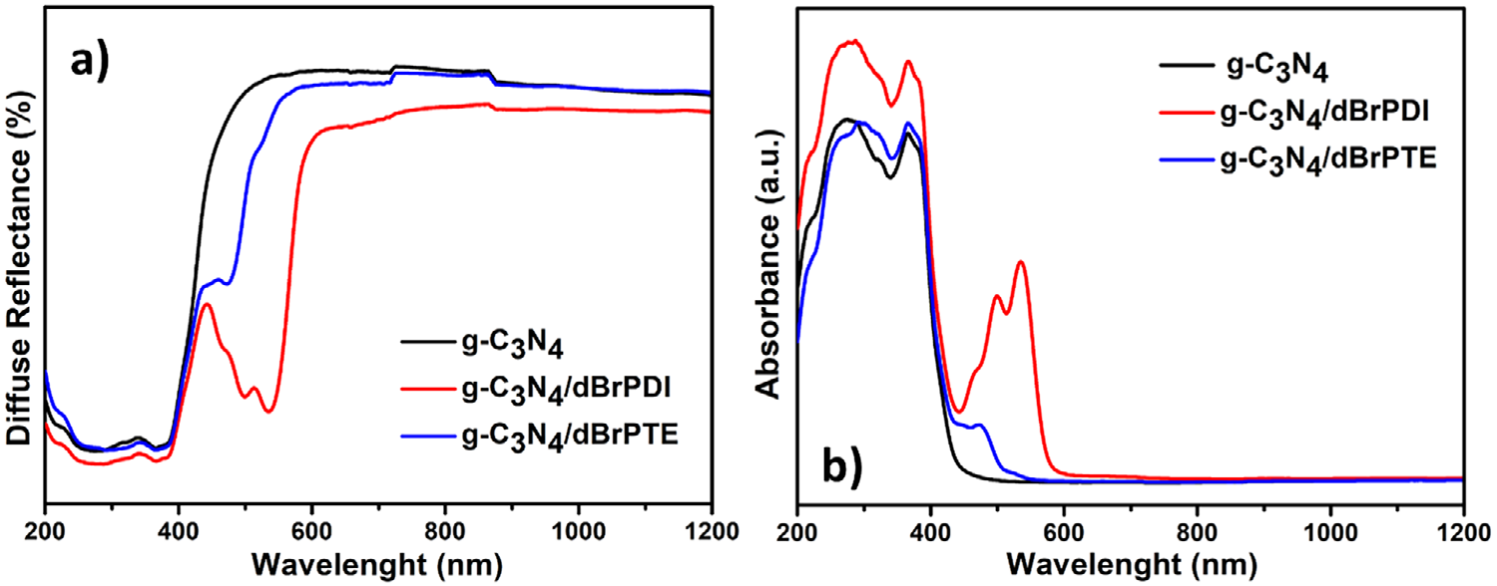
Optical properties of the as‐synthesized powders. Optical properties of the as‐synthesized powders. a) The diffused reflectance spectra of g‐C_3_N_4_, g‐C_3_N_4_/dBrPDI, and g‐C_3_N_4_/dBrPTE and b) UV–Vis–NIR absorbance spectra of g‐C_3_N_4_, g‐C_3_N_4_/dBrPDI, and g‐C_3_N_4_/dBrPTE by utilizing the Kubelka–Munk (K–M) reflectance theory.

The optical characteristics of the pure g‐C_3_N_4_, g‐C_3_N_4_/dBrPDI and g‐C_3_N_4_/dBrPTE are demonstrated in **Figure** **6**. The absorption spectrum curve in Figure 6b derived from transforming the diffused reflectance spectra of solid forms of nanoparticles into the absorbance by utilizing the Kubelka–Munk (K–M) reflectance theory.^[^ ^80^, ^81^ ^]^ It is observed that light absorption in the pure g‐C_3_N_4_ nanoparticle starts at around 420 nm. Pronounced absorption in the range of 400–500 nm resulting from the presence of dBrPTE is observed in the absorption spectrum of g‐C_3_N_4_/dBrPTE hybrid nanocomposite. In g‐C_3_N_4_/dBrPDI hybrid nanocomposite, strong absorption is observed at wavelengths expanding up to about 600 nm with peaks at about 550 and 500 nm resulting from the presence of dBrPDI. The sensitization process with brominated perylene derivatives leads to intense absorptions of hybrid nanocomposites in the visible region, contributing to increased photocatalytic degradation rates, as will be discussed in subsequent sections. Furthermore, it is observed that sensitizing with dBrPDI dye causes an increase in the UV region for hybrid nanocomposite. The light absorption spectrum corresponding to hybrid nanocomposites, including the visible region, represents a significant advantage for photocatalytic applications due to the fact that ≈44% of the sunlight spectrum constitutes the visible region.

X‐ray photoelectron spectroscopy (XPS) analyses were performed to further illustrate the chemical state of the g‐C_3_N_4_, g‐C_3_N_4_/dBrPDI, and g‐C_3_N_4_/dBrPTE hybrid nanocomposites. Figure 6a presents the XPS survey spectra  results of samples and the corresponding high‐resolution analysis results. The obtained XPS spectra show typical photoelectron peaks for g‐C_3_N_4_ at binding energies of 288 eV (C1s), 399 eV (N1s), and 532 eV (O1s).^[^ ^82^ ^]^ In the high‐resolution C 1s XPS spectra of the samples shown in Figure 6b, two peaks are observed at about 284.6 and 287.9 eV. The C1s peak at about 284.6 eV is typically associated with the sp^2^ C*─*N peak, while the peak at about 287.9 eV corresponds to C═O bonding.^[^ ^83^, ^84^ ^]^ The high‐resolution spectra of  N1s spectrum in Figure 6c can be deconvoluted into four peaks. The most intense peak observed is at 398.4 eV, corresponding to sp^2^‐hybridized nitrogen C═N*─*C. The nitrogen in N−(C)₃, which is tertiary N bonded to carbon in g‐C_3_N_4_, and the uncondensed terminal amino groups C*─*N*─*H are located at 399.18 and 400.9 eV, respectively.^[^ ^85^ ^]^ The weakest broad peak at 404.79 eV is attributed to charge effects (π‐excitons) or positive charge localization in heterocycles.^[^ ^3^, ^86^ ^]^ The O1s XPS spectrum of samples presented in 6d shows a main peak centered at about 532.14 eV, attributed to sp^2^‐hybridized O atoms in C═O ascribing to the surface hydroxyl groups.^[^ ^87^ ^]^

When the XPS spectra of g‐C_3_N_4_/dBrPDI and g‐C_3_N_4_/dBrPTE particles, sensitized with perylene dyes or decorated on g‐C_3_N_4_ via π–π interactions, were examined, no significant shift in the main peak energy positions of any element was observed. It is thought that the absence of peak shift detectable in the XPS spectra is due to the trace amount of perylene derivatives in the hybrid nanocomposites. That approximation is consistent with published reports.^[^ ^88^, ^89^ ^]^ It is seen from our implemented photocatalytic activities that the trace amount of perylene derivatives in the hybrid nanocomposites of g‐C_3_N_4_/dBrPDI, and g‐C_3_N_4_/dBrPTE can result in greatly facilitate the electron migration based on the π–π interaction between perylene derivatives and g‐C_3_N_4_ nanosheets through the electron delocalization effect.

The reactive oxygen species (ROS)^[^ ^90^ ^]^ produced by photoinduced electrons (e−) – holes (h+) pairs in the presence of photocatalyst, are formed in the process of advanced oxidation processes (AOPs). In the heterogeneous photocatalytic process, the generated reactive oxygen species, such as hydrogen peroxide (H_2_O_2_), singlet oxygen (^1^O_2_), superoxide anion radical (^•^O_2_^−^), and hydroxyl radical (^•^OH), give rise to the degradation of the pollutants in the aquatic media by transforming it into less harmful or harmless products.^[^ ^91^ ^]^

The degradation rate of molecules in the solution can be determined by tracking changes in their light absorption over time. According to the Beer–Lambert Law, the absorption of light realized by the homogeneous compound in the solution depends on its absorptivity and the concentration's ratio. During photocatalytic reactions under solar simulator irradiation, the maximum measured absorbance value gradually decreases as the molecules's concentration decreases due to molecular degradation. Assuming that the maximum absorbance value is directly proportional to its concentration in the solution, the degradation rate of molecules over time can be easily calculated using the Beer–Lambert Law, expressed by the following equation ^[^ ^92^ ^]^

(1) A=ε1c

where *A* is absorbance, *ε* is absorption coefficient, *l* is the length of the beam, and *c* is the concentration of the light‐absorbing molecule in the medium.

Reactive oxygen species (ROS) are key intermediates in the oxidative degradation of organic contaminants via heterogeneous photocatalysis and are widely employed in advanced oxidation processes for environmental remediation. Upon photoexcitation of a semiconductor, electron–hole pairs are generated, with electrons (e⁻) promoted to the conduction band (CB) and holes (h⁺) left in the valence band (VB). These charge carriers participate in redox reactions with molecular oxygen and water at the catalyst surface, leading to the formation of various ROS, including singlet oxygen (¹O₂), superoxide anion radicals (•O₂⁻), hydrogen peroxide (H₂O₂), and hydroxyl radicals (•OH).^[^ ^93^ ^]^ The intimate interfacial interaction and well‐aligned band structures between g‐C₃N₄ and perylene derivatives enhance interfacial charge transfer, specifically enabling the migration of photoexcited electrons from the conduction band (CB) of g‐C₃N₄ to the valence band (VB) of the perylene derivatives.^[^ ^94^ ^]^ The photogenerated electrons residing in the conduction band (CB) of the oxidation photocatalyst (OP) and the holes in the valence band (VB) of the reduction photocatalyst (RP) exhibit a propensity to recombine at the heterointerface, primarily driven by Coulombic interactions between oppositely charged carriers. Collectively, the synergistic effects of the internal electric field, interfacial band bending, and electrostatic (Coulombic) attraction serve as the principal driving forces facilitating the interfacial recombination of CB electrons from the OP and VB holes from the RP.^[^ ^95^, ^96^ ^]^ Within these architectures, an internal electric field (E) is established at the heterointerface, which significantly enhances photogenerated charge carriers' spatial separation and directional migration, thereby improving interfacial charge dynamics and overall photocatalytic efficiency.^[^ ^97^ ^]^ This band alignment, characterized by appropriately positioned conduction and valence band edges, provides a strong thermodynamic driving force for redox reactions. Consequently, the photogenerated charge carriers are efficiently scavenged by H₂O/OH⁻ and O₂, leading to the generation of highly reactive hydroxyl (·OH) and superoxide (·O₂⁻) radicals.^[^ ^98^ ^]^ These species exhibit high oxidative potential and low selectivity, enabling them to nonspecifically attack and cleave covalent bonds within organic molecules, ultimately mineralizing them into CO₂ and H₂O.^[^ ^99^ ^]^ The generic stepwise reaction pathways are presented below^[^ ^100^ ^]^

(2) $$h\upsilon \;\to \;e^{-}+h^{+}$$

(3) $$h^{+}+H_{2}O\left(\mathrm{ads}\right)\to HO^{•}\left(\mathrm{ads}\right)+H^{+}\left(\mathrm{ads}\right)$$

(4) $$O_{2}+2e^{-}\to {O_{2}}^{-•}\left(\mathrm{ads}\right)$$

(5) $${O_{2}}^{-•}\left(\mathrm{ads}\right)+H^{+}↔H{O^{•}}_{2}\left(\mathrm{ads}\right)$$

(6) $$H{O^{•}}_{2}\left(\mathrm{ads}\right)\to H_{2}O_{2}\left(\mathrm{ads}\right)+O_{2}$$

(7) $$H_{2}O_{2}\left(\mathrm{ads}\right)\to 2HO^{•}\left(\mathrm{ads}\right)$$

(8) $$HO^{•}+\mathrm{dye}\;\to \mathrm{intermediates}\;\to CO_{2}+H_{2}O$$

**Figure 7:**
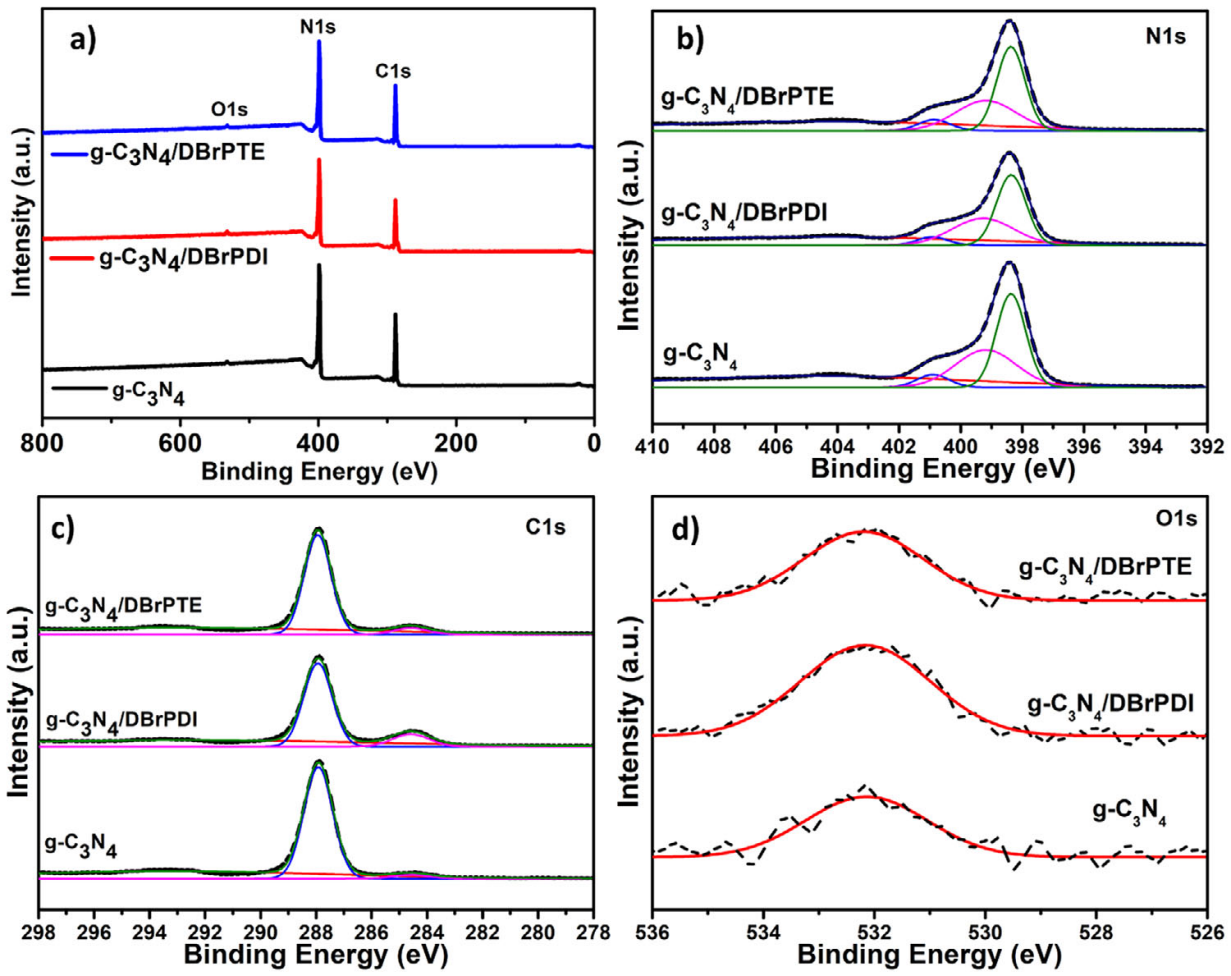
XPS survey spectrum a) and the corresponding high‐resolution spectra of N1s b), C1s c), O1s d) that are obtained from the g‐C_3_N_4_ nanosheets, g‐C_3_N_4_/dBrPDI, and g‐C_3_N_4_/dBrPTE nanocomposites.

**Figure 8:**
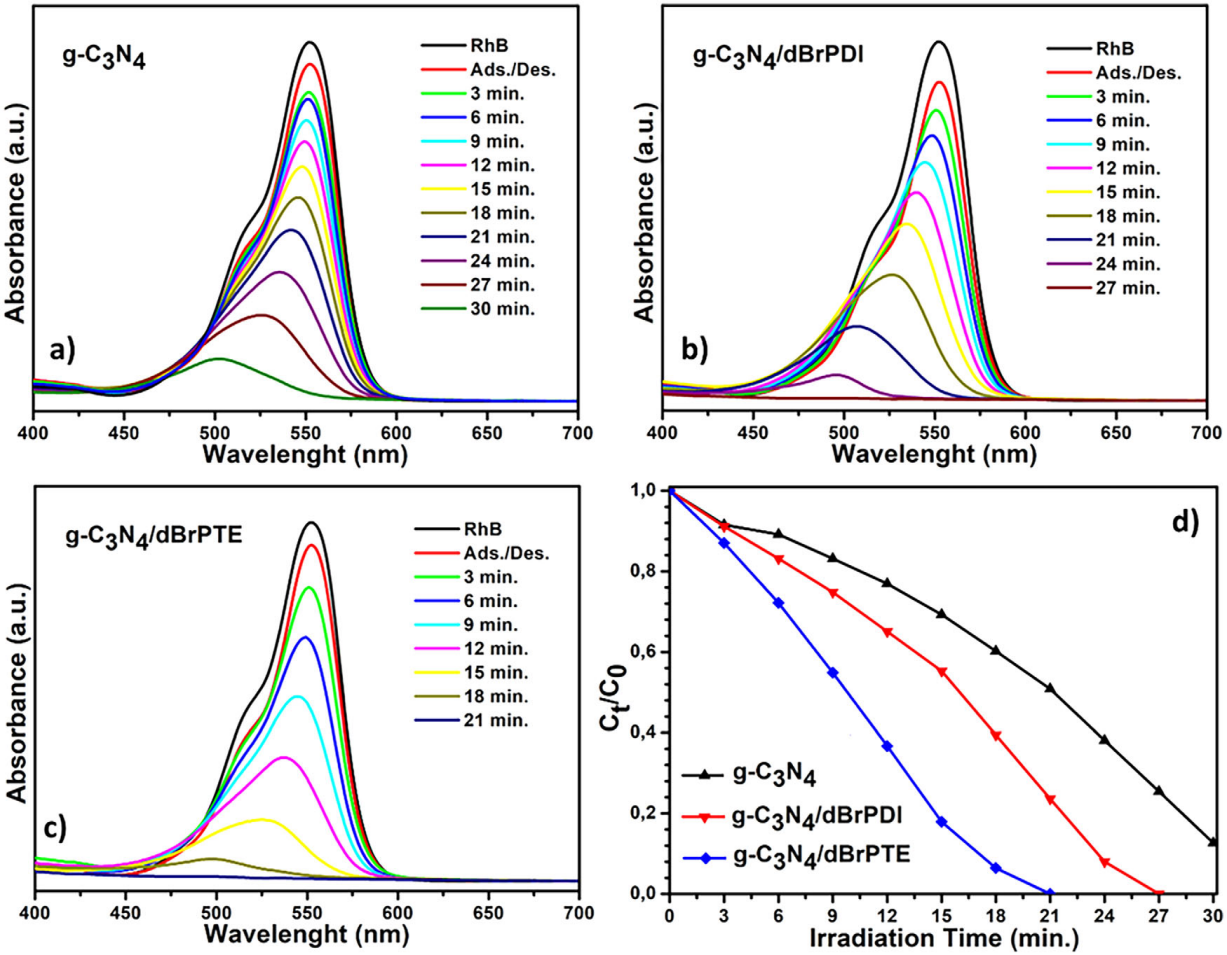
Photocatalytic activities of the as‐synthesized photocatalysts against model pollutant cationic dye rhodamine B under AM 1.5 G solar simulator. a) UV–vis absorption spectra of g‐C_3_N_4_. b) UV–vis absorption spectra of g‐C_3_N_4_/dBrPDI nanocomposite. c) UV–vis absorption spectra of g‐C_3_N_4_/dBrPTE nanocomposites and d) Transformed time‐concentration graph of photocatalysis test results calculated by the Beer–Lambert Law.

**Figure** **8** represents the results of absorption spectrum changes photocatalyzed by g‐C_3_N_4_, g‐C_3_N_4_/dBrPDI, and g‐C_3_N_4_/dBrPTE against rhodamine B (RhB) and their transformed concentration versus time graphs. As seen in **Figure** **7**a, the pure g‐C_3_N_4_ obtained by thermal exfoliation degraded ≈87% of the RhB within 30 min. The g‐C_3_N_4_/dBrPDI hybrid nanocomposites completely degraded RhB in 27 min (Figure 7b), while the g‐C_3_N_4_/dBrPTE hybrid composite achieved total degrading of RhB in only 21 min (Figure 7c). The degradation rate of RhB by formed g‐C_3_N_4_/dBrPDI hybrid nanocomposite showed an ≈50% increase compared to pure g‐C_3_N_4_, whereas the degradation rate enhancement of RhB with g‐C_3_N_4_ sensitized by dBrPTE reached nearly 100%. As a result, sensitization of g‐C_3_N_4_ with dBrPTE dye enhanced the photocatalytic degradation of RhB than sensitization of g‐C_3_N_4_ with dBrPDI.

**Figure 9:**
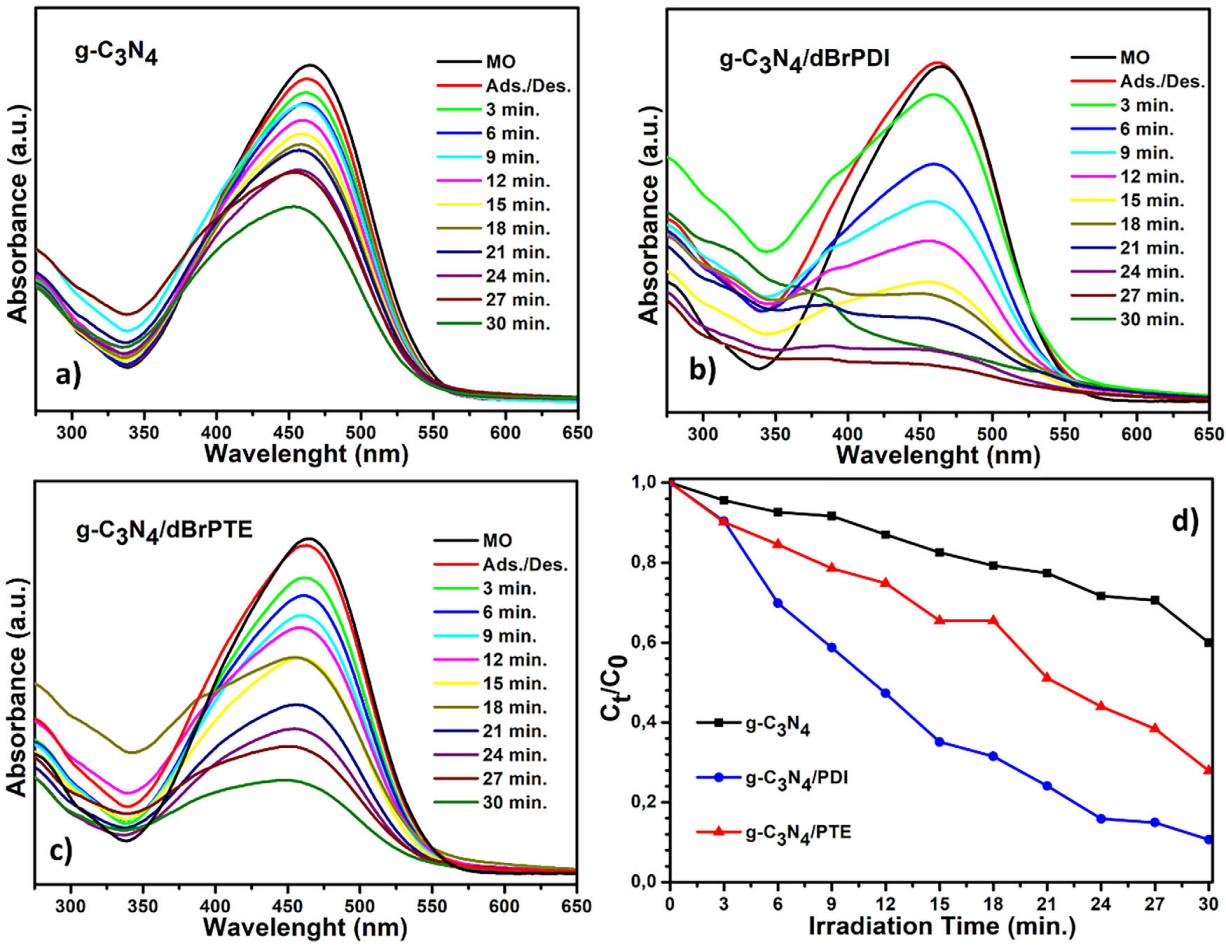
Photocatalytic activities of the as‐synthesized photocatalysts against model pollutant anionic dye methyl orange under AM 1.5 G solar simulator. a) UV–vis absorption spectra of g‐C_3_N_4_. b) UV–vis absorption spectra of g‐C_3_N_4_/dBrPDI nanocomposite. c) UV–vis absorption spectra of g‐C_3_N_4_/dBrPTE nanocomposites and d) Transformed time‐concentration graph of photocatalysis test results calculated by the Beer–Lambert Law.

**Figure** **9** represents the results of absorption spectrum changes photocatalyzed by g‐C_3_N_4_, g‐C_3_N_4_/dBrPDI, and g‐C_3_N_4_/dBrPTE against methyl orange (MO) and their transformed concentration versus time graphs. As seen in Figure 8a, the pure g‐C_3_N_4_ degraded about 40% of MO within 30 min. The g‐C_3_N_4_/dBrPDI hybrid nanocomposites degraded ≈90% of MO within the same time, while g‐C_3_N_4_/dBrPTE degraded around 72% within the same time. These results indicate that sensitization of g‐C_3_N_4_ nanocomposite photocatalysts with perylene dyes leads to improved photocatalytic efficiency for both. The hybrid nanocomposite sensitized with dBrPTE dye exhibits a degradation rate 1.8 times faster than pristine g‐C_3_N_4_, while the hybrid nanocomposite sensitized with dBrPDI dye shows a 2.25‐fold increase in degradation rate. Interestingly, while sensitising g‐C_3_N_4_ with dBrPDI exhibits the best photocatalytic performance in the photodegradation of MO, sensitising g‐C_3_N_4_ with dBrPDI shows the best performance in the photodegradation of RhB. In general, it is observed that a photocatalyst exhibiting a faster degradation performance against any pollutant dye exhibits a higher degradation rate compared to the other, even if the model pollutant dye is changed.^[^ ^101^, ^102^ ^]^ However, it is observed that this is different in this study. In the study conducted here, the degradation efficiencies of the sensitized hybrid nanocomposites under light are reversed when the ionic state of the pollutant model dye changes.

**Figure 10:**
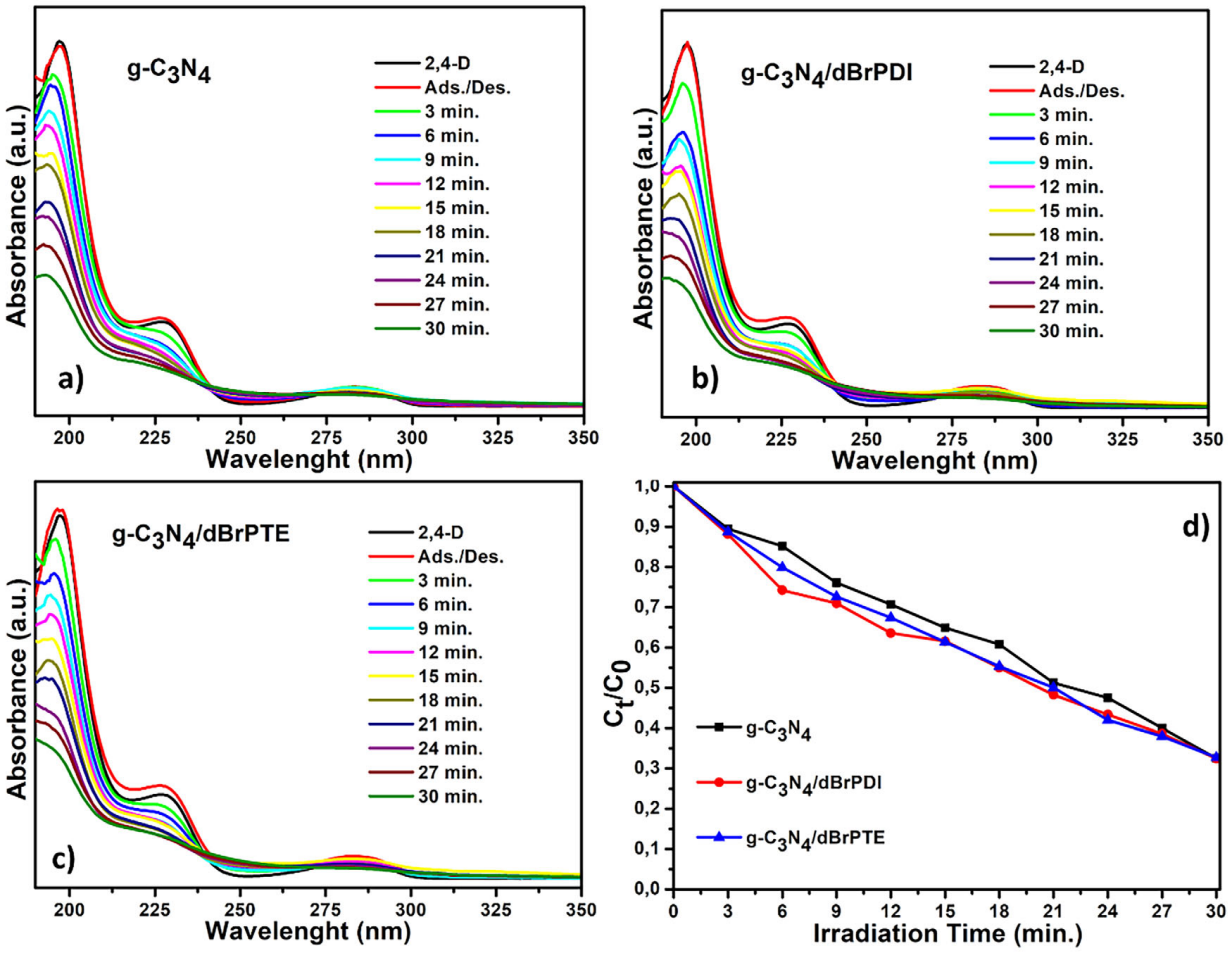
Photocatalytic activities of the as‐synthesized photocatalysts against model pollutant 2,4‐dichlorophenoxyacetic acid under AM 1.5 G solar simulator. a) UV–vis absorption spectra of g‐C_3_N_4_. b) UV–vis absorption spectra of g‐C_3_N_4_/dBrPDI nanocomposite. c) UV–vis absorption spectra of g‐C_3_N_4_/dBrPTE nanocomposites and d) Transformed time‐concentration graph of photocatalysis test results calculated by the Beer–Lambert Law.

The photocatalytic activities of the synthesized photocatalysts against 2,4‐dichlorophenoxyacetic acid (2,4‐D), selected as a model pollutant and one of the most widely used herbicides in agricultural activities, were also conducted under a solar simulator at the same conditions. The corresponding results are represented in **Figure** **10**, along with the depicted concentration versus time graph. As seen from the depicted concentration versus time graph in Figure 10d, the pure g‐C_3_N_4_, g‐C_3_N_4_/dBrPDI, and g‐C_3_N_4_/dBrPTE can degrade ≈68% of 2,4‐D over a 30‐min, which implies the same degradation rate for all photocatalysts. In contrast to the observed increases in efficiency for MO and RhB, no such increases are observed with the perylene dye sensitization in the photocatalytic degradation of 2,4‐D. In the case of sensitization with dBrPDI and dBrPTE, although an initial increase in the degradation rate of the photocatalytic degradation process is observed at the beginning, this rate increase diminishes over time, levelling off to match that of pristine g‐C_3_N_4_ (**Figure** **10**).

It is noteworthy to note the changes in nanocomposite photocatalyst effectiveness on the photocatalytic degradation of RhB and MO model dyes. While g‐C_3_N_4_/dBrPTE hybrid nanocomposite was superior against RhB degradation, g‐C_3_N_4_/dBrPDI hybrid nanocomposite performed better over MO degradation. The reversal in degradation efficiencies appears to be linked to the ionic state of the pollutant dyes. The observed results suggest that the ionic nature of the target molecule deeply affects charge transfer and charge separation mechanisms. It is thought that the electron–hole separation mechanism and photogenerated charge transfer mechanism in the perylene derivatives sensitized‐C_3_N_4_‐based photocatalysts are more complex and depend significantly on the ionic nature of the model pollutant molecule. It is also thought that the exceptional photocatalytic activity increase in the case of MO and RhB is mainly ascribed to the efficient S‐scheme charge transfer, and charge separation is supported via the π–π interactions, leading to more produced ROS.

**Figure 11:**
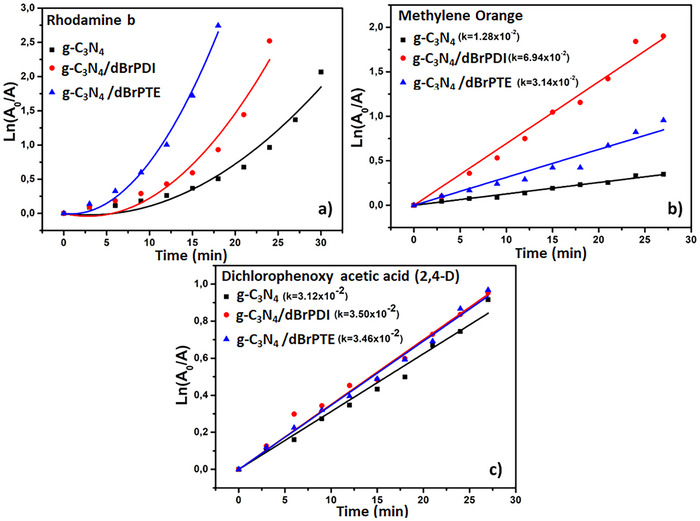
The second‐order photocatalytic degradation kinetics of a) RB and the first‐order degradation kinetics curves of b) MO and c) 2,4‐D are given.

In **Figure** **11**, the effects of g‐C_3_N_4_, g‐C_3_N_4_/dBrPDI, and g‐C_3_N_4_/dBrPTE photocatalysts on the degradation kinetics (*k*) of MO, RB, and 2,4‐D were investigated. The degradation kinetics were evaluated by plotting ln(*A*_0_/*A*) values ​​against time. In Figure 11a, the degradation kinetics were observed as second order, while in Figure 11b,c, it was first order.

The effects of different photocatalysts for each pollutant were summarized by calculating *k* constants and comparing them in **Table** **1**. For g‐C_3_N_4_, the rate constants on 2,4‐D, MO, and RhB were given as 3.13, 1.28, and 2.13 × 10^−2^ min^−1^, respectively. This shows that the activity of this photocatalyst on MO is lower compared to other pollutants. For g‐C_3_N_4_ /dBrPDI, these values ​​are 3.50, 6.94, and 3.14 × 10^−2^ min^−1^. It reveals that g‐C_3_N_4_ /dBrPDI shows a higher catalytic activity on MO. For g‐C_3_N_4_ /dBrPTE, these values ​​are 3.46, 3.38, and 7.33 × 10^−2^ min^−1^. It was found that the highest catalytic activity on RhB was achieved by g‐C_3_N_4_/dBrPTE.

**Table 1:** Catalysis rate constants (*k*) of g‐C_3_N_4_, g‐C_3_N_4_/dBrPDI, and g‐C_3_N_4_/dBrPTE catalysts used in the photocatalytic degradation of MO, RB, and 2,4‐D.

	Catalysis rate constants of photocatalysts [*10^−2^ dk^−1^]		
	**g‐C_3_N_4_**	**g‐C_3_N_4_/dBrPDI**:	**g‐C_3_N_4_/dBrPTE**
**2,4‐D**	3.13	3.50	3.46
**MO**	1.28	**6.94**	3.38
**RhB** ^a^ ^)^	2.13	3.14	**7.33**

^a)^ Calculated for the first 12 min.

Experimental findings reveal that g‐C_3_N_4_/dBrPDI exhibits the highest catalytic effect in the photocatalytic process of MO compared to other catalysts. Similarly, g‐C_3_N_4_/dBrPTE demonstrates superior photocatalytic performance in the degradation of RhB. On the other hand, while both dBrPDI and dBrPTE sensitized catalysts enhance the catalytic rate of 2,4‐D degradation compared to the reference (g‐C_3_N_4_), the increase is not as significant as observed in MO or RhB systems. These findings emphasize the critical importance of selecting an appropriate sensitizer based on the type of pollutant in degradation studies.

## 3 Conclusion

This study highlights the potential of metal‐free g‐C₃N₄ as a next‐generation visible light‐responsive photocatalyst, particularly when paired with perylene derivative nanocomposites, to enhance photocatalytic performance. By employing ultrasonic‐assisted self‐assembly, exfoliated g‐C₃N₄ was sensitized with brominated perylenediimide (dBrPDI) and perylene tetraester (dBrPTE) in toluene solution, resulting in improved light absorption, charge transfer, and charge separation. These advancements significantly increased efficiency in degrading organic pollutants, particularly RhB and MO model dyes. The study further revealed that the ionic nature of the target pollutant profoundly influences the photocatalytic degradation process. While the g‐C₃N₄/dBrPTE hybrid nanocomposite demonstrated superior activity in RhB degradation, the g‐C₃N₄/dBrPDI hybrid nanocomposite was more effective against MO degradation. These findings suggest that the charge transfer and separation mechanisms, supported by S‐scheme heterojunction and π–π interactions, are crucial in optimizing photocatalytic activity. The exceptional generation of reactive oxygen species (ROS) is attributed to these mechanisms, underscoring the complexity and dependency of photocatalytic efficiency on the pollutant's ionic state. This work provides valuable insights into the development of brominated perylenediimide and perylene tetraester modified g‐C₃N₄‐based S‐scheme nanocomposite photocatalysts, emphasizing their potential for efficient pollutant degradation in aquatic environments and contributing to addressing critical environmental challenges.

## 4 Experimental Section

### Materials and Instruments

Melamine, ethanol, hydrochloric acid (%37), bromine (Br_2_), hexane, chloroform, toluene, dichloromethane, *N*, *N*’‐dimethylformamide, acetic acid, 1‐methyl‐2‐pyrrolidinone, and silica gel (0.040–0.063 mm), thin‐layer chromatography (TLC) were sourced from Sigma‐Aldrich, while perylene‐3,4,9,10‐tetracarboxylic dianhydride (PTCDA) was acquired from Fluka.

### Synthesis of g‐C_3_N_4_ Nanosheets and Perylene Derivatives

The graphitic carbon nitride (g‐C_3_N_4_) nanosheets were produced in two steps by using a melamine precursor. The g‐C_3_N_4_ nanosheets were synthesized by thermal polymerization followed by thermal exfoliation. A detailed procedure for synthesizing g‐C_3_N_4_ nanosheets can be found in the previous work.^[^ ^70^ ^]^ Synthetic details of 1,7‐dibromo perylene‐3,4,9,10‐tetracarboxylic acid butyl ester (DBrPTE) and *N*,*N*′‐bis(2‐ethylhexyl)‐1,7‐dibromo perylene‐3,4,9,10‐tetracarboxylic diimide (DBrPDI‐2EH) used in this study can be found in the previous work.^[^ ^64^, ^71^ ^]^

### Preparation of g‐C_3_N_4_ Sensitized with dBrPDI or dBrPTE

The dBrPDI and dBrPTE sensitized g‐C_3_N_4_ hybrid nanocomposites are synthesized through ultrasonic assisted self‐assembly technique. 50 mg of g‐C_3_N_4_ nanosheets and 15 mg of dBrPDI or dBrPTE are separately added into 50 mL of toluene. The mixture is then subjected to ultrasonic treatment for 45 min. Subsequently, it is filtered through standard filter paper, and the solid is dried in a vacuum oven at 60 °C throughout the night.

### Characterizations Technics

Phase characterization of samples was conducted by using a RIGAKU SmartLab XRD operated at 40 mA current and 40 kV voltage with Cu‐Kα radiation (*λ* = 1.5406 Å).

Microstructural and chemical properties were carried out by using a field emission gun TESCAN MAIA XMU scanning electron microscope (FE‐SEM).

The optical analysis and photocatalytic performances of samples were executed by using a Shimadzu UV‐3600 UV–Vis–NIR spectrophotometer.

Surface area analyses of the synthesized pure g‐C_3_N_4_ and g‐C_3_N_4_ hybrid nanocomposites sensitized with perylene derivatives are carried out by BET nitrogen adsorption–desorption. The determination of the specific surface area of ​​the prepared samples is carried out at 77 K using Quantachrome ASiQwin instrument after the sample is degassed under vacuum at 378 K. Fourier‐transform infrared (FTIR) spectra of the samples are obtained using a IRAffinity‐1S FTIR Spectrophotometer (Shimadzu) in the range between 4000 and 700 cm^−1^.

### Photocatalytic Activity Measurements

Photocatalytic activity experiments of g‐C_3_N_4_ nanosheets, dBrPDI and dBrPTE sensitized g‐C_3_N_4_ hybrid nanocomposite photocatalysts against anionic methyl orange (MO), cationic rhodamine B (RhB), dyes, and 2,4‐dichlorophenoxyacetic acid (2,4‐D) herbicide were carried out under atmospheric conditions. The model pollutant solutions to be used in photocatalytic studies were prepared as 10 mg L^−1^ for all model pollutants. Each photocatalytic studies were carried out by adding 25 mg photocatalyst into 50 mL of model pollutant solution. The suspension containing photocatalyst was continuously stirred with a magnetic stirrer in the dark for 60 min to ensure the adsorption–desorption equilibrium on the surface of the particles before placed under the solar simulator. Then, the dispersion was placed under an AM 1.5 solar simulator and excited with a 500 W xenon lamp under continuous magnetic stirring conditions. After establishing adsorption–desorption equilibrium in the dark, 3 mL samples were taken from the solution to determine the adsorption characteristics of the nanoparticles. 3 mL samples were taken from the solution at 3‐min intervals to determine the photocatalytic degradation rate resulting from exposed light irradiation for each photocatalytic experiment. In all photocatalytic tests, samples taken from the suspensions were centrifugated to separate the catalyst nanoparticles from the aquatic medium. To monitor the photocatalytic performances of the synthesized nanoparticle photocatalysts against model pollutant molecules, the UV–vis–NIR absorption spectra of the samples at 3‐min intervals were characterized using a UV–vis–NIR spectrophotometer.

## Conflict of Interest

The authors declare no conflict of interest.

## Data Availability

The data that support the findings of this study are available from the corresponding author upon reasonable request.
